# Vector Disparity Sensor with Vergence Control for Active Vision Systems

**DOI:** 10.3390/s120201771

**Published:** 2012-02-09

**Authors:** Francisco Barranco, Javier Diaz, Agostino Gibaldi, Silvio P. Sabatini, Eduardo Ros

**Affiliations:** 1 Department of Computer Architecture and Technology, CITIC, ETSIIT, University of Granada, C/Daniel Saucedo Aranda s/n, E18071, Granada, Spain; E-Mails: jdiaz@atc.ugr.es (J.D.); eduardo@atc.ugr.es (E.R.); 2 PSPC Group, Department of Biophysical and Electronic Engineering (DIBE), University of Genoa, Via Opera Pia 11A, I-16145, Genoa, Italy; E-Mails: agostino.gibaldi@unige.it (A.G.); silvio.sabatini@unige.it (S.P.S.)

**Keywords:** field programmable gate arrays, active vision, real time systems

## Abstract

This paper presents an architecture for computing vector disparity for active vision systems as used on robotics applications. The control of the vergence angle of a binocular system allows us to efficiently explore dynamic environments, but requires a generalization of the disparity computation with respect to a static camera setup, where the disparity is strictly 1-D after the image rectification. The interaction between vision and motor control allows us to develop an active sensor that achieves high accuracy of the disparity computation around the fixation point, and fast reaction time for the vergence control. In this contribution, we address the development of a real-time architecture for vector disparity computation using an FPGA device. We implement the disparity unit and the control module for vergence, version, and tilt to determine the fixation point. In addition, two on-chip different alternatives for the vector disparity engines are discussed based on the luminance (gradient-based) and phase information of the binocular images. The multiscale versions of these engines are able to estimate the vector disparity up to 32 fps on VGA resolution images with very good accuracy as shown using benchmark sequences with known ground-truth. The performances in terms of frame-rate, resource utilization, and accuracy of the presented approaches are discussed. On the basis of these results, our study indicates that the gradient-based approach leads to the best trade-off choice for the integration with the active vision system.

## 1. Introduction

Depth perception is essential for an autonomous system that is moving in a dynamic environment. It is applied in multiple applications such as autonomous navigation [1], obstacle detection and avoidance [2], 3-D reconstruction [3], tracking [4,5], grasping [6,7], *etc*. For example, depth computation is used for obtaining 3D information of real-world scenarios with real-time implementations such as [8,9]. Many computer vision algorithms have been proposed for extracting depth information from multiple-view images [10,11].

**Figure 1.**
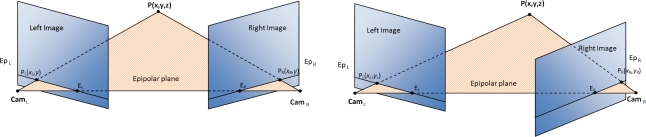
Epipolar geometry for a pair of cameras. **Left:** *P* corresponds to (*x_L_, y*) and (*x_R_, y*) coordinates for the left and right image planes respectively. **Right:** The same *P* point corresponds to different *x* and *y* coordinates on each image plane. *Ep_L_* and *Ep_R_* are the epipolar lines, *Cam_L_* and *Cam_R_*, the camera optical centers, and *E_R_* and *E_L_* stand for the epipoles.

An alternative for depth estimation is based on the correspondences of image features of a binocular system. Disparity computation models basically consist of a matching problem, finding correspondences between features or areas in both left and right images and taking into account some constraints based on the geometry of the stereo rig that may help to simplify the search process (due to the epipolar geometry constraint [10]). This model assumes that the geometry of the cameras is fixed and known (e.g., using a previous calibration stage that allows us to extract the intrinsic and extrinsic binocular cameras parameters [10]). Based on this assumption, in most of the literature contributions, the disparity is managed as a mono-dimensional matching problem of correspondences. They usually assume that images are calibrated or include a pre-processing stage for undistortion and rectification. This process consists of correcting the lens radial distortions and aligning the image planes with the epipolar lines, which simplifies the disparity estimation, reducing the problem to a matching only for the horizontal coordinates (see Figure 1). Nevertheless, the main problem is that the rectification process is required each time the camera configuration is modified. Therefore, this technique is not suitable for any active system that is able to modify the vergence of the cameras, which means that we need a different approach.

In our case, we select a system that adaptively controls the vergence of its binocular camera setup. The use of a static vergence angle cannot yield to a correct gazing position to a specific point. However, a solution for this problem is to use a vergence control that allows the modification of the relative position of the cameras in order to fixate a point. This bio-inspired solution achieves the best disparity results around the fixation point because it moves the image plane at the depth of this point and computes a very small range of disparities around it. In general, indeed, the highest accuracy of the disparity calculation kernel is obtained with the lowest disparity ranges.

On the other hand, our active binocular sensor acts depending on the target, which means that its fixation point is determined by three parameters: version, tilt, and vergence angles (see Section 2). There are some previous works that support the use of the disparity for guiding vergence eye movements in active vision systems [12–14]. The listed advantages allow us to explore the scene in detail, by changing gazing and fixation at different targets or areas of interest. In this work, we present the implementation of a system where vision and motion control act in a collaborative manner for solving the presented issue. With respect to the motion control strategy, our work is based on a separate control of version and vergence inspired by the Hering’s law [15]. Hering proposed that the movement of one eye is coordinated with the other with a movement of equal amplitude and velocity, but it could be in opposite directions. This coordinated movements are specified in terms of version and vergence components. However, eyes only approximate this law, because saccadic movements may include additional vergence components [16]. More details are provided in the following subsection.

In the literature, we find different works that deal with the vector disparity estimation problem [17–19]. In this work, we propose a comparison between a gradient-based [20,21] and a phase-based [22–24] approach for the disparity computation. Phase-based approaches are more accurate and robust against variations in the illumination. On the other hand, gradient-based approaches may be implemented at a reduced cost, saving resources in comparison with the first one and presenting a good accuracy.

FPGA is selected as the platform for our system because of the requirements of real-time performances and the suitability of the integration with the active vision system due to the reduced chip size and limited power consumption compared with other approaches as the one based on GPUs. For vision processing algorithms with a high-computational complexity, we need to exploit the maximum parallelism at different levels and in our case, this objective is matched using a fine-pipeline based architecture in an FPGA. In the case of the single-scale version of our disparity computation, we achieve up to 267 and 118 fps (frames per second) for the gradient- and the phase-based algorithms, respectively. For the multi-scale implementations, the reached frame rate is almost 32 fps. In both cases, the image resolution is VGA (640 × 480), although higher resolutions are possible at the cost of reducing the frame rate.

This paper is structured as follows: in Section 2, we present the vergence, tilt, and version control for our sensor; in Section 3, we describe the mathematical formulation of the used approaches for the disparity computation (gradient-based and phase-based); Section 4 analyzes the hardware implementations for both gradient-based and phase-based approaches detailing their mono- and multi-scale versions. This section also presents the performance analysis (accuracy and frame rate) and the resource utilization. Section 5 shows the motion control strategy for our binocular system and the integration of the system with the disparity computation. Finally, Section 6 presents the conclusions and the future work.

## 2. Version, Tilt, and Vergence Control

In this section, we present a system that manages the vergence, version, and tilt control with a stereo pair of cameras to fixate a target object. This is possible thanks to the utilization of a vector disparity model and a camera setup endowed with a shared motor for the tilt control, and two motors for the independent control of the pan angle of each camera. The separate controls allow us to move the gaze toward the point of interest and to control the vergence angle to fixate the target.

**Figure 2.**
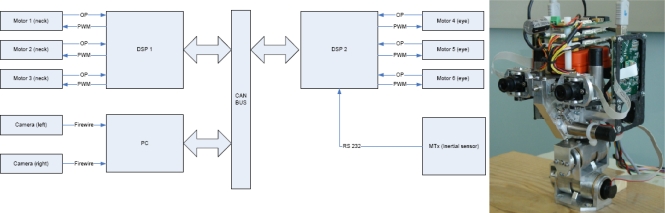
Hardware architecture of the iCub head. On the left, the architecture with the two DSPs, the PC connected to the CAN bus, and the inertial sensor are shown. An image of the head is shown on the right.

The experiments described in Section 5 were performed using the iCub head platform designed by the RobotCub Consortium [25]. Figure 2 displays the real RobotCub head and the architecture scheme. This platform is composed by a stereo pair of FireWire cameras, two DSPs, and an inertial sensor connected to a PC. The communication between the elements and the PC is carried out through a CAN bus. The DSP controllers use Motorola 56F807 16-bit hybrid processors. The head consists of a binocular system with six degrees of freedom: the eyes have two rotational degrees of freedom but with a common tilt; the neck also has three degrees of freedom. The control of cameras and neck motors is performed separately by two DSP units. The main problem with such a platform is the sensitivity of the motors to the low velocities, or different friction for the motors which might cause oscillations in the fixation task. Finally, slower responses of the system yield more predictable results. More information about the mechanics is provided in [26,27]. Finally, cameras provide pairs of 15 fps with a 1,024 × 768 resolution, although we are able to compute up to 32 fps of 640 × 480 pixels of resolution, we crop and subsample the resolution to 320 × 240 for our experiments because it provides good results for the vergence control algorithm. Our disparity computation system is based on an adaptable architecture that allows us to set the image resolution using input parameters. In this way, it is possible to adopt the most appropriate resolution for the target application. A smaller resolution for the images in our experiments allows the tracking of the objects in a very controlled scenario, and helps us to avoid any possible problem that may be caused by the rapid movements of the head.

**Figure 3.**
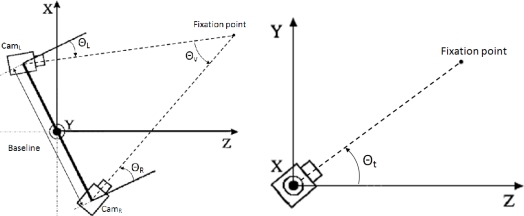
Version, vergence, and tilt angles for the stereo vision system. Top and side views of the camera configuration showing the version, vergence, and tilt angles for each camera.

The voluntary fixation of the visual system is directly related with the fovea, the central area of the retinas, that provides high-resolution visual stimuli. In fact, in human vision, fixation movements have the task to register the target into the foveae, in order to maximize the perceptible details in the area of interest [28]. Version is defined as the rotation of the eyes about the vertical axis to maintain a constant disparity. Meanwhile, tilt is the rotation of each eye with respect to the horizontal axis. Finally, vergence is the rotation of each eye about the vertical axis to change the disparity. These parameterization of the system allows to define a unique fixation point with three angles [29]. In Figure 3, we observe the top and side views of our camera setup. In the figure, Θ*_v_* defines the vergence angle, Θ*_L_* and Θ*_R_* stand for the pan angles (version) and finally, Θ*_t_* is the tilt angle (displayed on the right). As we mentioned before, tilt angles are common for both right and left cameras. Due to the correlation between the angles, if the vergence angle is defined, we only need either Θ*_L_* or Θ*_R_* to define version.

Version might be determined monocularly using a master eye (camera in our case) for the gazing to the target object and the slave camera performs a vergence movement to fixate the point. In this case, we only have two separate controls. However, vergence has to be defined in terms of binocular disparity.

The selected joint control method combines version, vergence, and tilt as independent parallel movements. The performed control can be expressed by the following set of equations in Equation (1).

(1) $$\begin{array}{lll} \Theta_{\mathrm{version}} & = & K_{1}(x_{L}+x_{R})\\ \;\;\;\;\;\;\;\;\Theta_{\mathrm{tilt}} & = & K_{2}(y_{L}+y_{R})\\ \Theta_{\mathrm{vergence}} & = & K_{3}\;f(d) \end{array}$$

where (**x**_*L*_, **y**_*L*_) and (**x***_R_*, **y***_R_*) are the coordinates of the target for the left and right images regarding the current fixation and *f*(**d**) is a function of the disparity for the fixation point for almost similar vertical disparities that only avoids nullifying the vergence angle for the zero disparity and gives the disparity in other case. Finally, *K*_1_, *K*_2_ and *K*_3_ are tuning gains. The area close to the fixation point is defined as “zero disparity” region. Vergence control allows us to fixate the gazed target by searching for this region.

We assume the computation of real-time disparity for the implementation of our control model. A fast vergence control can be computed using the information of the disparity of the target when the gazing is changing continuously, and our system is trying to reduce it to “zero” at this point.

There are also some different alternatives such as performing the fixation with several fixed setups for the cameras, constraining them to a set of more likely depths for the fixation point. This approach seems to save resources, since it avoids the computation of the real vector disparity (the undistortion and rectification stage has to be done, but the implementation is easy since the setups are fixed and known “a priori”). As our approach is also applied to autonomous mobile vehicles, vibrations may affect the setup, therefore a periodic evaluation of the rectification would be needed. From this perspective, the strategy of fixed setups is unsuccessful, since the rectification steps keep being mandatory. Another alternative may consist of using several fixed cameras with different vergence configurations, completely avoiding the rectification stage. This approach is similar to the previous one but it entails a high economic cost, which makes it unsuitable for a lot of applications.

## 3. Comparison of Vector Disparity Algorithms

The binocular disparity is usually defined as the difference in the *x* coordinates between the right and left images of a binocular vision system. This definition is useful because, even if in real-world applications the images are uncalibrated, binocular disparity systems usually include a preprocessing rectification stage that compensates for the difference in the *y* coordinate. This operation is related to the geometry of the cameras, and needs to be recomputed each time that the relative position of the cameras is modified. If the rectification is not applied (for instance, because the relative position of the cameras is continuously changing as in active vision systems), the disparity becomes a 2D problem, as we will discuss in the next subsections.

Disparity estimation techniques can be grouped into local and global methods. Local methods are centered in the surrounding neighborhood of a pixel to estimate its disparity. Global methods take into account the complete image. In our case we implement two different alternatives: a gradient-based technique, the well-known local algorithm of Lucas and Kanade [20,21] and a phase-based one detailed in [23] (also a local algorithm). The first technique estimates small local disparities assuming the intensity or brightness constancy of a pixel between left and right images, while the second one computes the disparity using the phase information for different orientations, in a contrast-independent way. In order to increase the working range for disparity detection, we use the multi-scale extension of the algorithms. Both implementations have been developed from the horizontal-disparity implementations (1D) but conveniently extended for the vector disparity (2D).

In order to validate our approach, we compare both the methods in terms of their different advantages and drawbacks. While the gradient-based method provides a good trade-off between efficiency and resource utilization, phase-based methods are very robust against variations in illumination and shadows, which makes them specially appropriate for real-world applications. In this paper, we avoid the use of global algorithms because they are not suitable for the on-chip implementation, at least with an affordable resource cost. This also explains why most of the current embedded implementations of disparity computation engines are based on local approaches [28,30–34].

### 3.1. Gradient-Based Lucas–Kanade Disparity Model

The disparity is defined as a 2-D problem as shown in Equation (2):

(2) $$I^{\mathrm{right}}(x,y)=I^{\mathrm{left}}(x+d_{x},y+d_{y})$$

where *I^right^* and *I^left^* are the intensity of right and left images respectively and *d_x_* and *d_y_* are the disparity components. The solution of the system in this case is similar to the case of the optical flow in [35,36]. Applying the Taylor expansion in Equation (2), we obtain Equation (3)

(3) $$d_{x}I_{x}^{\mathrm{left}}+d_{y}I_{y}^{\mathrm{left}}+I^{\mathrm{left}}(x,y)-I^{\mathrm{right}}(x,y)=0$$

where $I_{x}^{\mathrm{left}}$ and $I_{y}^{\mathrm{left}}$ are the partial derivatives of the left image. With this ill-posed system, additional assumptions need to be considered. The Lucas–Kanade algorithm supposes that pixels in the same neighborhood correspond to the same object and therefore, have a similar disparity. And then, applying a least-square fitting for solving Equation (4) procedure, we obtain the system defined by Equations (5) and (6).

(4) $$\left(d_{x},d_{y}\right)={\left(A^{T}W^{2}A\right)}^{-1}A^{T}W^{2}b$$

(5) $$(A^{T}W^{2}b)=\left[\begin{array}{l} \sum_{i\in \Omega }W_{i}^{2}I_{\mathrm{xi}}^{\mathrm{left}}\left(I_{i}^{\mathrm{left}}-I_{i}^{\mathrm{right}}\right)\\ \sum_{i\in \Omega }W_{i}^{2}I_{\mathrm{yi}}^{\mathrm{left}}\left(I_{i}^{\mathrm{left}}-I_{i}^{\mathrm{right}}\right) \end{array}\right]$$

(6) $$(A^{T}W^{2}A)=\left[\begin{array}{ll} \sum_{i\in \Omega }W_{i}^{2}{\left(I_{\mathrm{xi}}^{\mathrm{left}}\right)}^{2} & \sum_{i\in \Omega }W_{i}^{2}I_{\mathrm{xi}}^{\mathrm{left}}I_{\mathrm{yi}}^{\mathrm{left}}\\ \sum_{i\in \Omega }W_{i}^{2}I_{\mathrm{xi}}^{\mathrm{left}}I_{\mathrm{yi}}^{\mathrm{left}} & \sum_{i\in \Omega }W_{i}^{2}{\left(I_{\mathrm{yi}}^{\mathrm{left}}\right)}^{2} \end{array}\right]$$

where *W_i_* stands for the weighting matrix of the pixels in the neighborhood Ω. For the optical flow, Barron [20] computes the confidence of the computed estimation thresholding with the minimum of the eigenvalues of Matrix (6). We simplify it by using the determinant of this Matrix without a significant loss of accuracy, as shown in [36,37]. Finally, this algorithm also provides very computationally efficient solutions with a competitive accuracy as shown in [38,39].

### 3.2. Phase-Based Disparity Algorithm

Sabatini *et al*. proposed in [23] a multi-channel interaction algorithm to combine the phase information from multiple spatial scales (the multi-scale approach will be detailed in Section 3) and multiple orientations. The computation is performed combining multiple Gabor filter responses tuned at 8 orientations. Using the same formulation for Gabor filtering of [40], the vector disparity can be computed from the phase difference applying an intersection of constraint along different orientations, as in [41], assuming that points on an equi-phase contour satisfy *ϕ*(*x, t*) = *c*, with *c* a constant. Differentiation with respect to time yields Equation (7):

(7) ∇ϕ⋅d+ψ=0

where $\nabla \phi ={\left(\frac{\delta \phi }{\delta x},\;\frac{\delta \phi }{\delta y}\right)}^{T}$ is the spatial phase gradient, *d* = (*d_x_, d_y_*)*^T^* is the vector disparity, and *ψ* is the phase difference between left and right images. The phase difference is computed without any explicit phase computation using the formulation proposed in [24]. In a linear model, the spatial gradient can be substituted by the radial frequency vector (*w*_0_*cosθ_q_, w*_0_*sinθ_q_*) [23] where *q* indicates one of the eight orientations of the Gabor filter bank that we use. Next, Equation (7) can be rewritten as Equation (8)

(8) $$w_{0}({\cos\theta }_{q},{\sin\theta }_{q})\cdot d=-\psi_{q}$$

where · denotes scalar product. From this point, we can extract the component disparity and finally compute the disparity estimation solving the over-determined system defined in Equation (9)

(9) $$d_{x}(x)w_{0}\cos\theta_{q}+d_{y}(x)w_{0}\sin\theta_{q}+\psi_{q}(x)=0$$

### 3.3. Multi-Scale Generalization

As mentioned, our development is based on the multi-scalar generalization or coarse-to-fine scheme, inspired on Bergen’s work [42], that increases the working range 30 times with respect to the mono-scalar implementation (using 5 scales). Moreover, it is a mandatory operation to achieve fully-operative systems on real-world scenarios. Our architecture is based on warping images, an approach that is usually avoided in the real-time embedded system literature because of its high resource costs (although it is more cost-effective than global methods).

The multi-scalar version implementation is simple. Firstly, we compute the pyramid for the input images (left and right) and they are stored (see [43]). In such a way, we have a bank of images sampled at different spatial resolutions (depending on the number of scales). The second step consists of iterating in a loop as many times as scales: for each iteration, we upsample the previous disparity estimation; then, we warp the previous upsampled results and the frames for the following finer scale; next, the new disparity estimation is computed using the previous results as input for the core; the final stage collects the new estimation and the partial previous, stored results to combine them in a new partial estimation for the next iteration. The first iteration only consists of the initialization, computes the estimation using as input the images computed in the pyramid for the first spatial resolution scale, and continues to the next iteration.

## 4. Hardware Implementation

The selected device for our hardware implementation is an FPGA. As mentioned in the introduction, the FPGA is a good candidate as a platform that allows us to exploit the maximum level of parallelism to achieve an embedded system able to work in real-time. Furthermore, the second key point is the possibility of integration with the motor control for our binocular system. The selected FPGA is a Xilinx Virtex4 chip (XC4vfx100). The board is a Xirca V4 [44] with a PCIe interface and four SRAM ZBT memory banks of 8 MB each one. This platform can work as a stand-alone platform or a co-processing board. This means that the platform can be used separately working alone or connected with a PC which facilitates the hardware implementation debugging and the result display.

Our super-scalar architecture was developed using fine-pipelined datapaths. The implementation of this ILP (Instruction Level Parallelism) provides high performances and low power consumption [36]. Our architecture is displayed as an adaptable design for different applications and requirements as is shown in some previous works [30,35,36,45]. The use of fixed-point arithmetic entails a high resource saving with an acceptable loss of accuracy (provided that the bit-width at different operations is carefully chosen).

Our design validation consists of evaluating the accuracy of this degradation with respect to a software floating-point version. We split the complete processing engine into different stages and test different bit-widths for the variables at each stage. The key point is to find the minimum bit-width that leads to a minimum loss of accuracy. Once our objective is reached, we continue with the following stage sequentially along the datapath. This process has been successfully adopted in other contributions as [30,35,36] and represents a good working methodology to allow the fixed-point implementation of floating point algorithms using digital hardware.

The development of the implementation was performed using two hardware description languages for two different abstraction levels. Firstly, the implementation of the modules that perform the communication protocols and interfaces and the Memory Controller Unit (MCU [46]) are implemented in VHDL. The disparity computation and the multi-scale architecture was developed using Handel-C because this C-like language is better suited for algorithmic descriptions, without significantly degrading performances or increasing resources [47].

The hardware implementation of the multiscale extension is described in detail [35,40,45]. This multi-scale-with-warping architecture is usually avoided in the literature because of the high computational costs that imply in hardware implementations high resource costs. On the other hand, the multi-scale architecture that we implemented allows us to increase the working range 30 times with respect to the mono-scale implementation.

### 4.1. Lucas–Kanade Vector Disparity Core

The implementation of this core is based on previous approaches [35,36,48]. In the cited works, the authors implement optical flow gradient-based algorithms, for both mono- and multi-scale versions. In this work, the most important difference is the computation of the vector disparity or bidimensional disparity for uncalibrated images instead of the 1-D disparity for rectified and undistorted images.

**Figure 4.**
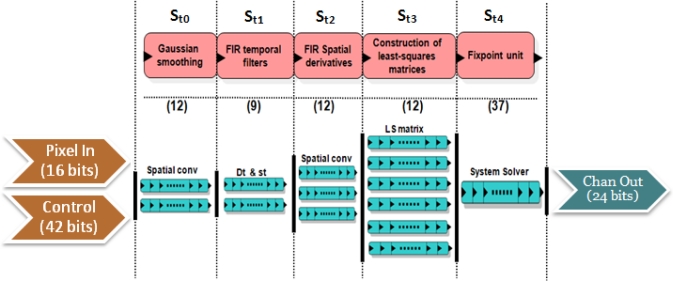
Scheme of the pipelined stages for the Lucas–Kanade vector disparity core. It describes the computation stages (from *St*_0_ to *St*_4_) indicating the pipelined stages (in brackets) and the number of parallel datapaths for each one of them.

In Figure 4, *Pixel In* denotes the input to the disparity core (in our case, 2 frames for the left and right images with a bitwidth of 8); *Control* represents the control word, with the parameters for the number of scales, confidence thresholds, and the input resolution. The output bitwidth is 24 bits: 12 bits for the vertical and the horizontal component of the disparity. The core is implemented in a segmented pipeline design with 5 stages:

- *St*_0_: In this stage, the filtering of the inputs reducing the aliasing effects is performed. It convolves them with Gaussian filters whose kernels of 3 taps are *K* = [1 2 1]/4.
- *St*_1_: It computes the left-right difference and applies a smoothing filter to the inputs.
- *St*_2_: In this stage, we compute the spatial derivatives to the results of the previous stage: *I_x_*, *I_y_*, and filter again the results (including the left-right difference).
- *St*_3_: This stage performs the calculation of the coefficients for the linear systems of 5 and 6. The weights *W* are set to a 5 × 5 separable kernel defined *W* = [ 1 4 6 4 1]/16 as in [36,37].
- *St*_4_: The final stage computes the solution of the system using the previous results. It uses as confidence measure the determinant of this matrix.

For the computation of this core, we use 187 parallel processing units: stage *St*_0_ has 2 paths (2 frames) for the Gaussian convolution, *St*_1_ has 2 paths (for the left-right difference and the smoothing), *St*_2_ has 3 paths (2 for the derivatives *I_x_* and *I_y_* and 1 for the left-right difference), *St*_3_ has 6 paths (one for each coefficient in Equations (5) and (6)) and *St*_5_, has only one path for the system resolution. The number in brackets in the figure denotes the micropipelined stages for each of them.

### 4.2. Phase-Based Vector Disparity Core

The design of this core is also based on a previous approach [30]. The main difference with respect to our case is that previous approaches implement a core for the computation of disparity for calibrated images, as in the previous core (as in [30,34]).

**Figure 5.**
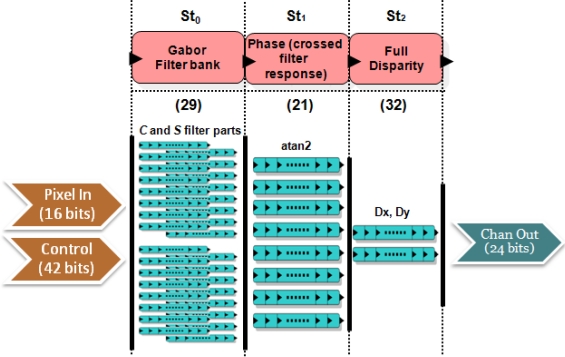
Scheme of the pipelined stages for the phase-based vector disparity core. It describes the computation stages (from *St*_0_ to *St*_2_) indicating the pipelined stages (in brackets) and the number of parallel datapaths for each one of them.

In Figure 5, we show a scheme of the design. Input parameters are the same as in the case of the previous core: *Pixel In* denotes the input data (2 frames for left and right images with 8 bits); *Control* for the control word (42 bits). The output bitwidth is 24 bits: 12 bits for each disparity component. In this case, the core is implemented in a segmented pipeline with 3 stages:

- *St*_0_: This stage computes the odd and even filtering quadrature components for the image pair.
- *St*_1_: It computes the disparity for each orientation (we use 8 orientations).
- *St*_2_: The provided component disparities need to be combined to compute the final full disparity. They conform an equation system solved in this stage applying least-squares (see Equation (9)).

For the computation of this core, we use 1,160 parallel processing units: stage *St*_0_ has 32 paths (2 frames) for the computation of the odd and even filter components (with 8 orientations, we have 16 different filters), *St*_1_ has 8 paths for the *atan2* operations and finally, *St*_2_ has 2 paths, one for each disparity component. The number in brackets in the figure denotes the micropipelined stages for each of them. We have used two IP cores from Xilinx Core Generator platform to compute complex arithmetic operations such as arctangent (*St*_1_) and a pipelined division (*St*_2_).

### 4.3. Multi-Scale Architecture

The implementation of the multi-scale architecture allows us the expansion of the working range of our disparity estimation more than 30× (using 5 scales) compared to the case of mono-scale versions. The design of this multi-scale architecture is the same as in previous works [30,45]. This approach is inspired by Bergen’s work [42].

**Figure 6.**
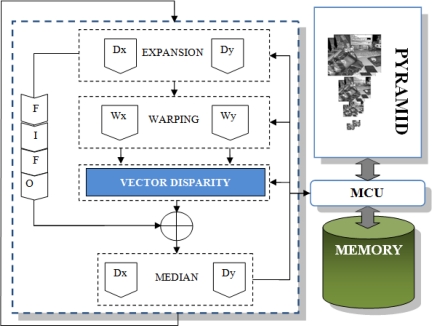
Hardware system architecture. Right side: the pyramid and its communication with memory. Left side: multi-scale computation (scaling, warping, merging, median filtering and vector disparity computation).

The multi-scalar version implementation is simple and it is displayed in Figure 6. Firstly, we compute the pyramid for the input images (left and right) and store them. In such a way, we have a bank of images sampled at the different spatial resolutions (depending on the number of scales). The second step consists of iterating in a loop as many times as scales: for each iteration, we upsample the previous estimation of the disparity; the next stage consists of the computation of the warping using the previous up-sampled results and the frames for the following finer scale; the warping operation compensates (removes) the apparent movement of scene elements due to the different points of view, allowing us the computation of disparity estimates in a valid range for that scale; the new disparity estimation is computed using the previous results as input for the core; the final stage collects both the new estimation and the partial previous stored results, combining them in a new partial estimation for the next iteration. The first iteration only consists of the initialization and computes the estimation using as input the images computed in the pyramid for the first spatial resolution scale and goes to the next iteration.

As a brief summary of this architecture, the design is divided into the following modules:

- Gaussian pyramid: This module implements the computation of the Gaussian pyramid of the left and right images for the disparity computation inspired in [43]. The number of levels of this pyramid depends on the number of scales with a downsampling factor of 2. It is built by a smoothing and a subsampling circuit. The main operations at this step are the 2D convolution with Gaussian filters of 5 taps (smoothing) and the sub-sampling. The kernel is a 5-by-5 matrix decomposed in two arrays *K* = [ 1 4 6 4 1]/16. Thanks to the use of separable filters, the convolution is performed in a parallel way for the *x* and *y* operations. Five image lines are stored in an embedded multi-port BlockRAM used like a FIFO for the convolution. Then, we send to the output (the external SRAM) a pixel every two clock cycles: one pixel is discarded (sub-sampling). This sequential part of the processing is performed once and the results are stored/read to/from the RAM memory banks.
- Vector Disparity: This module has been explained in detail in previous subsections. Depending on the case, this module implements the vector disparity estimation based on the Lucas–Kanade or the phase-based algorithm.
- Expansion: This module up-samples the current scale results to the resolution of the following finer scale (the up-sampling factor is also 2). In contrast with the mono-dimensional disparity, this module is duplicated for the vector disparity. We use one module for each component of the vector disparity that needs to be up-sampled to the next scale resolution.
- Warping: In the literature, this computationally expensive stage is usually avoided because of its high computational cost. In our case, it consists of a bilinear interpolation between left and right image (keeping the left frame and warping the right one with the estimation of the disparity computed for the previous scale). In Equation (10), the warping computation for the vector disparity computation is shown. Θ denotes the bidimensional scaling operator for **d**(*x, y*) (vector disparity) at the scale *s* with a factor of 2 and *I^R^* stands for the right image.
  (10) $$I_{R}^{s}=\mathrm{Warp}(I_{R}^{s-1}(x,y),\Theta (d^{s-1}(x,y)))$$
  The warping operation for the vector disparity consists of a bilinear interpolation of the input images with the shift values which we have stored from the computed disparity in the previous scale. The computation of each warped pixel requires the reading of the pair (Δ*x*, Δ*y*) and the correspondent pixel *P*. Each pair (Δ*x*, Δ*y*) is used for retrieving from memory the four pixels of the original image for each *P* pixel. Then, the warped pixel is calculated performing a weighted bilinear interpolation with the obtained values. The warping process needs to perform four memory accesses per clock cycle to calculate one warped pixel to achieve the maximum throughput. This is one of the reasons for the choice of a specific MCU to manage data with different Abstract Access Ports (AAP) [46]. The warping architecture uses a reading AAP of the MCU for accessing the original image. The MCU provides a 36-bit bus allowing the access to four pixels per memory read. The X and Y matrices are provided from the expansion circuit through two blocking FIFOs. Warping requires a neighborhood of 4 pixels and the number of data available per memory access is limited to four in a same line. Thus, one access brings 2 pixels of the same line in the best case. Nevertheless, it is impossible to access the 4-pixel window in a single memory access. In the worst case, we access 4 different memory words (every 4 consecutive memory accesses). Therefore, the performance is constrained up to 4 pixels every 10 memory accesses.
- Merging: This module computes the addition of the previous feature estimation and the current one. The result is stored for the next iteration. The non-valid values are propagated from the coarsest scales to the finest ones. At the last scale, the finest one, we make the logical AND operation between its non-valid values and the propagated ones for the final estimation (the non-valid values obtained at the finest scale are the most reliable). The main problem of this module is the synchronization between the current and the stored results.
- Homogenization: This stage filters the results for each iteration with two 3 × 3 median filters in cascade. This filtering removes non-reliable values and homogenizes the results.

More details about the hardware implementation of this architecture can be found in [35,40,45].

## 5. Discussion of Results

As explained in the Introduction, with an active sensor that changes the vergence, the rectification process is required each time that the camera configuration is modified. This fact makes the static rectification approach unsuitable for this kind of systems. Moreover, an adaptive vergence system allows gazing on a target or fixation point and it may obtain the optimal disparity estimations around the image plane that is set using the depth of that fixation point. This section analyzes the performances of the vector disparity computation and the comparison in resource costs between our system and the same system with the static rectification unit.

We present the comparison of the developed systems in terms of resource utilization, accuracy and density of the results. Firstly, we list the performances of our work and some state-of-the-art publications with different implementations and technologies. Next, we benchmark our work (both approaches) with the set of images of Middlebury [49]. All this dataset is addressed to work with horizontal disparity algorithms; therefore, it is only shown to illustrate the comparison of our 2D disparity performances with the 1D ones. As mentioned before, our approach is suitable for working with active vision systems. We also test our implementations with our own benchmark images especially plotted to test the vector disparity accuracy and some images from an on-line set of benchmarks available at [50]. The benchmarking is performed for the hardware and software approaches to compare also the accuracy degradation due to the fixed-point arithmetic adopted in the hardware implementation. The last part of the section is dedicated to present a summary of the resource utilization.

In the case of the mono-dimensional benchmarks, we compute the MAE (Mean Absolute Error) and the Density. We also compute the PoBP (percentage of bad pixels, *i.e.*, the percentage of pixels whose MAE is greater than 1, see [40]). For the vector disparity estimation, we compute the AAE (Average Angular Error), and the Density. Furthermore, we also compute the PoGP (percentage of good pixels, or the percentage of pixels whose AAE is less than 5, see again [40]).

### 5.1. State-of-the-Art Comparison

We mentioned in the Introduction that our aim is the development of a high-performance system that works in real time (in our case, it means a frame rate of at least 25 fps with VGA resolution). In Table 1, we find that our implementation reaches up to 32 fps with a resolution of 640 × 480. This result fulfills our requirements.

Table 1 shows a performance comparison between our four developments (mono- and multi-scale approaches, phase-based, and Lucas–Kanade) with the last works in the literature. Due to the lack of works for computing vector disparity, we summarize in the table the most important ones that compute horizontal disparity in the last years. Besides, the mono-scalar version achieves a frame rate calculated using the maximum working frequency. Finally, in the multi-scalar versions, the frame rate is empirically measured using the proposed architecture and taking into account the PCIe bandwidth restrictions for the communication with a PC.

Most of the listed works in Table 1 are mono-scalar developments except [30] and [17]. The performances depend on the algorithm, the architecture, and the optimization level. Algorithms with a lower computational cost as SAD-based implementations may obtain better speed performances but the accuracy results are also rather low. Furthermore, there are substantial differences between mono- and multi-scalar versions. On the other hand, we merge horizontal and vector disparity algorithms in the same table to easily compare the main performances, but this comparison is not completely fair, e.g., in the case of the PDS. The PDS (Points × Disparity measures per Second) is a metric that measures the number of operations performed by our algorithm per time taking into account the disparity range. With respect to this last measure, Georgoulas *et al*. [28] achieves impressive results with a PDS of 21120 × 10^6^. Our PDS for the best multi-scalar version is 7122 × 10^6^. The computation of the PDS depends on the disparity range and, for vector disparity algorithms, this range is squared compared with the horizontal disparity range. In the column, we show firstly the PDS using the simple range to check the magnitude differences with the 1D algorithms and the second values are the PDS for the 2D implementation.

To estimate the disparity at different spatial resolution levels, the multi-scalar architecture is revealed as essential. The Laplacian pyramidal approach for the multi-scale architecture [43] is the way to implement this multi-resolution analysis through a fine-to-coarse strategy. The objective is to estimate disparities larger than the filter support. The factor depends on the number of scales (determined by the target application). In the case of the mono-scalar versions, the main advantage is obviously a computation with a high frame rate. Moreover, multi-scalar versions obtain significant precision improvements due to its wide-range estimations.

### 5.2. Benchmarking for Horizontal Disparity

In this subsection, we briefly analyze the performances of our vector disparity algorithms with the well-known benchmark images of Middelbury [49], whose vertical disparity components are always zero. As vector disparity implementations are rare in the literature, we include this section for future comparisons with horizontal disparity algorithms. Actually, we do not expect better results than the proper 1D algorithms, any variation in the vertical disparity (that should be zero) adds error to the resolution of the 2D equation system. Moreover, the search space for the solution is developed from 1D to 2D (see Equations (3) and (9) respectively) which makes the solutions more unreliable.

**Figure 7.**
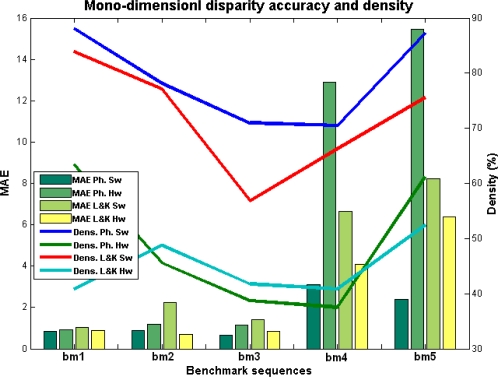
Horizontal disparity comparison: Lucas–Kanade *vs.* Phase-based and Hardware *vs.* Software versions (MAE and density).

Figure 7 and Table 2 show the performance analysis for the horizontal disparity computation. In the literature, besides the example which is being used in this paper, there are not many image benchmarks for vector disparity. Most of the works which we find in the literature test their algorithms and implementations with the classical horizontal disparity benchmark provided by Middlebury [49]. Firstly, we also show the efficiency and density of our approaches with some images of this benchmark, in particular: “*Tsukuba*”, “*Sawtooth*”, “*Venus*”, “*Teddy*”, and “*Cones*” (respectively called *bm*1 to *bm*5). We distinguish between the Lukas–Kanade and the phase-based version and between the hardware and the software implementations. In the figure, we notice that the error is similar for the different versions except in the case of *bm*4 and *bm*5 (“*Teddy*” and “*Cones*” cases). The increment in these cases of the errors for the phase-based hardware version is substantial and also entails an important loss of density. The Lucas–Kanade hardware version achieves even better results than the software version due to the use of homogenization stages. The table shows the best results for the Lucas–Kanade hardware version but, except for the last two cases, all the results are very similar and the degradation of the precision is not quite significant.

The generalized loss of density is very significant and may be attributed to the loss of precision of both designs. But it is even worst in the case of the phase-based implementation constrained for the fixed-point arithmetic, especially in the complex operations such as divisions and arctangent modules and in computations for the Gabor filtering stage. Moreover, the use of the fixed-point arithmetic also affects the computation of the confidence measure that performs the thresholding operation. Finally, it is also worth regarding that hardware implementations in Figure 7 are generally more accurate than their correspondent software implementations (more accurate and reliable estimations entail discarding more estimations).

### 5.3. Benchmarking for Vector Disparity

**Figure 8.**
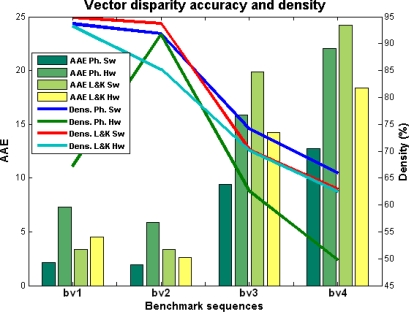
Vector disparity comparison: Lucas–Kanade *vs.* Phase-based and Hardware *vs.* Software versions (AAE and density).

In this last subsection, we perform the benchmarking using the convenient set of images for the analysis for the performances of the vector disparity implementations. Figure 8 and Table 3 show the vector disparity results for the “*plane 0H 0V*”, “*plane 15H 15V*”, “*desktop00*”, and “*desktop09*” sequences (marked as *bv*1 to *bv*4). The first two sequences (*bv1* and *bv2*) were generated using an initial ground-truth, whereas *bv*3 and *bv*4 are active views of 3D acquired natural scenes generated by a virtual reality system [50] and available at [56]. In this case, differences between the software and hardware versions are more important. On the other hand, the differences between the phase-based and Lucas–Kanade have been shortened, although the Lucas–Kanade hardware implementation seems slightly better. This fact can be attributed, as in the previous section, to the loss of accuracy that affects the phase-based model due to the bit-widths and fixed-point arithmetic limitations. Table 3 also supports it, showing a PoGP that model is about 25% higher in the Lucas–Kanade than in the phase-based model.

In general, in the case of this benchmark, the density is quite similar in contrast with the previous subsection. The computation is not affected as dramatically as in the previous case because now we are using appropriately the confidence measure (implemented for the 2D model not for the 1D). For instance (except for the first sequence *bv*1), the maximum difference between hardware and software results in density is about 15%, with similar tendencies for all the sequences.

**Figure 9.**
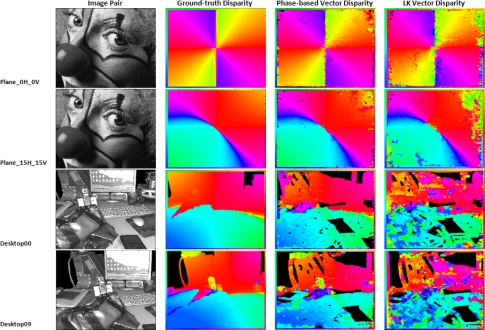
Software benchmark results for vector disparity. From left to right: original image, ground-truth, software results for phase-based, and Lucas–Kanade algorithms. The frame codes the vector disparity with a color.

**Figure 10.**
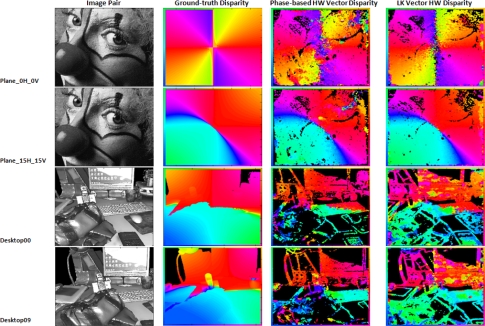
Hardware benchmark results for vector disparity. From left to right: original image, ground-truth, hardware results for phase-based, and Lucas–Kanade algorithms. The frame codes the vector disparity with a color.

Finally, Figures 9 and 10 show the disparity results for the software and hardware versions for the sequences available at [56] and the ones that we generated. In the figures, we display the original images, the ground-truth, and the results for the software versions in the case of the first figure and the hardware results for the second one. All the estimations have a color-coding frame describing the direction of the estimated vector disparity. The values in black in the hardware column are the unreliable values (*NaN*). As it can be seen, the phase-based results present better results in the case of the software but, for the hardware implementation, the bit-width constraining degrades the final estimation in an appreciable way. On the other hand, the phase-based implementation presents some advantages for its use in real-world applications such as a better behavior against variations in the illumination [57,58]. This last advantage is illustrated in the figures, especially for “*desktop00*” and “*desktop09*”. For a more detailed study of the robustness of phase-based implementations against illumination changes and affine transformations between the stereo pair images, cf. [57,59,60].

### 5.4. Hardware Resource Utilization

In computer vision, the selection of the best alternative is performed depending on the target application, searching for a good trade-off between the required accuracy (see previous subsections) and constraints about the maximum frequency and the resource utilization. Table 4 shows the resource utilization and the maximum working frequency of the implemented system. This table presents the information about the board resource utilization: total number of 4 input LUTs, Slice Flip Flops, Slices, DSPs, Block RAMs used, and finally, the maximum frequency (MHz).

The listed options include: the multi-scale vector disparity systems and the horizontal disparity systems with the rectification stage, for both approaches. More details about the resource cost of the different unit components of the multi-scale generalization can be found at [30,35,45].

For the horizontal disparity systems, the Lucas–Kanade version uses about 9% more 4-Input LUTs, this is due to the difference in Block RAMs and DSPs and especially, to the optimization level. Tools for hardware implementations apply optimization techniques based on heuristics that cannot be completely controlled using the software parameters. A totally fair comparison can also be done with a more intensive use of resources.

On the other hand, focusing on the vector disparity computations, while the phase-based approach increases the resource utilization about 10%, the Lucas–Kanade approach achieves a remarkable reduction of about 25%. As the multi-scale architecture is the same for both approaches, the increment in resource costs for the phase-based approach is due to the 2D core. While the rectification unit is the bottle-neck for the Lucas–Kanade architecture, it is the proper 2D core for the phase-based scheme.

All these results support the idea of implementing the final system for motion control of our binocular system with the Lucas–Kanade approach taking into account also the low degradation of accuracy obtained in the previous subsection and the resource saving of more than 25% with respect to the phase-based approach.

## 6. Control of Vergence for a Binocular System

As described in the Introduction section, our final objective is the implementation of a system that, using the vector disparity (implemented according to the previous sections), manages the vergence, version, and tilt control.

Motor control is distributed using Digital Signal Processing (DSPs) boards, in this case, Freescale DSP-56F807, 80 MHz, fixed point 16 bits which perform a fast low-level control loop in real time. A CAN-bus line allows the communication between the boards and a remote PC. Motors are directly controlled by standard PID controllers. This DSP, due to its memory and computation limitations, implements simple operations such as pre-filtering, signal acquisition, and PID position control (using absolute position encoders). More details about the mechanics and the architecture of the ICub and RobotCub can be found in [26,27].

The algorithm that we use for the fixation consists of selecting a point of our object of interest and computing a centroid based on the color of this point, using a threshold for the RGB components (a range of [*−*5,5] for each component). Once we have the centroid computed, we firstly compute the distance between this point and the current position of the eye centers and send it via the CAN bus to the DSP-based control cards. The communication protocol allows four working modes: sending relative angles, absolute angles, relative pixel positions, or normalized relative position (see the documentation at [25]); as explained, we use the third alternative. The PID low-level control loop acts on the motors to modify version and tilt according to these coordinates of the color-based centroid and in this way, we set our eye centers in the object of interest. The last step is acting on the vergence motors and computing the vector disparity until it reaches a value near zero (we use a threshold of 5). Changing the vergence of the cameras means translating the image plane to the depth of the target. Once the object is fixated, we will obtain the optimal performances around this object (zero disparity plane).

Tracking adds the possibility of performing a smooth pursuit of the object gazing on several fixation points. It is worth noticing that, due to the huge backlash on the eyes pan movement [25] and the limits in the physical properties of the motors, the pursuit is not as smooth as it may be expected and there are some fluctuations shown in the second experiment.

One of the problems that might appear in this scenario is the motion blur between the left and right camera images. In our algorithm, we firstly have a master camera that moves to the color centroid (where the object of interest is) and fixates to this position, followed by the second camera. As explained, the tilt is common to the cameras, while the version is achieved to move the cameras towards the same orientation. Then, the second camera (the slave) achieves the vergence movement to fixate the object, according to the position of the first eye.

The blur may happen during this second step, since the disparity is now being computed. However, we partially avoid this situation by using the disparity after each movement when the rig setup of the cameras is steady and not constant. In addition, the multiscale process also provides robustness to this issue due to the coarse-to-fine process and the limited disparity range possible at each scale.

In this final section, we present the results for two real experiments: the fixation in a target object, in our case it is a red plastic bottle, and the fixation and tracking of a blue LED light.

### 6.1. Experiment 1: Red Plastic Bottle Fixation

**Figure 11.**
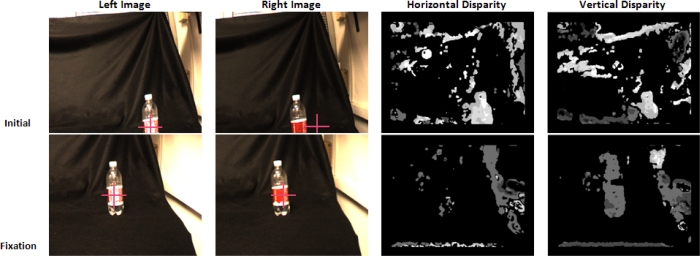
Initial position and fixation for the red plastic bottle example. Disparity components are displayed.

The first experiment consists of making the system fixate at a red plastic bottle. Figure 11 displays the initial position of the system (left and right images of the cameras). The red cross shows the fixation point in each case. The processing begins with the selection of the fixation point and then, the system performs the fixation task with the static object. In the figure, we observe the initial horizontal disparity at the fixation point; after fixating the point, the horizontal disparity tends to zero (we use a threshold to avoid variations that might affect the stability of the system). The vertical disparity is also almost zero, and in our case, with a common tilt for both cameras, it is not possible to reduce it.

**Figure 12.**
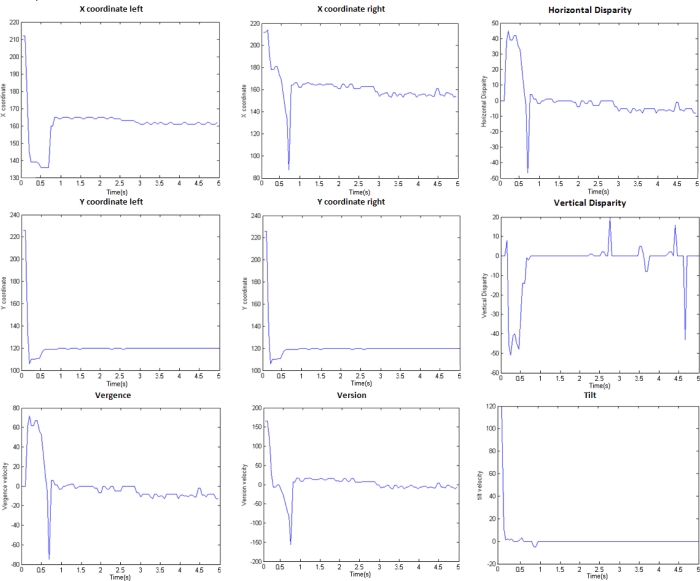
Red plastic bottle fixation example: X and Y coordinates for left and right images, horizontal and vertical component disparities and the evolution of version, vergence, and tilt velocities along the time (image resolution is 320 × 240).

The evolution of the X and Y coordinates of the fixation point for both cameras and the component disparities are plotted in Figure 12. As we see, with an image resolution of 320 × 240, the fixation is finally performed in a very accurate and fast way. After 0.8 s, all the evolutions of the different parameters are stabilized (X and Y coordinates at 160 and 120 values respectively; component disparities around zero). The third row shows the evolutions for the vergence, version, and tilt. We plot the velocities for the different parameters that are the inputs of the DSP that controls the different motors of our system. After 0.8 s, all of them are also stable. Similar results are obtained for fixation in several works using other architectures: in [61], the fixation time is about 0.5–0.6 s and in [62], about 1.5 s. Our approach is shown as a very competitive alternative according to the fixation time and it has been also presented as one the most accurate ones.

### 6.2. Experiment 2: Smooth Pursuit

**Figure 13.**
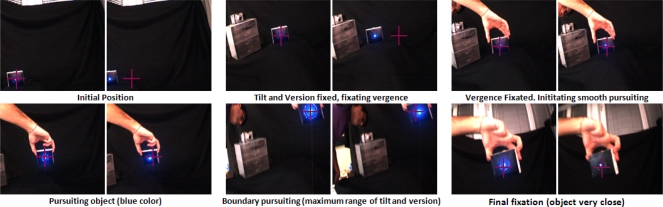
Blue LED smooth pursuit. At each different phase, we show the left and right images of our system, beginning with the fixation and then, with the pursuit of the target object.

In this experiment, we show the smooth pursuit example with a blue object. Figure 13 shows the experiment performed for the pursuit of a blue object. The sequence shows the initial fixation and some subsequent captures of the cameras of our system tracking the object even at the limits of its physical properties. Once the object is detected, the system always performs the fixation again.

It is worth noticing that the fixation point is not exactly the same point of the object in both cases, on the left and right camera images, because, as we mentioned, we use a threshold for the zero disparity region. In this way, our system is more stable and smoother trajectories are ensured.

## 7. Conclusions

The main contribution of this work is the implementation of a sensor for an active vision system with dynamic vergence control to explore dynamic environments using a scheme different to the common static camera configurations. The main problem of an active system is that the rectification process is required each time that the camera configuration is modified. Therefore, the use of a static rectification preprocessing stage technique is unsuitable for any active system that actively modifies the vergence angle of its camera setups. Our solution consists of using vector disparity estimations to control vergence. We have developed a SoC (system-on-a-chip) that integrates vector disparity estimation with vergence control on the same chip. This allows an embedded implementation with a low consumption at a single device that can be integrated on the i-Cub head (or any vision system including low level processing stages such as active stereo vision).

The first part of this work is dedicated to the design and implementation of an embedded system for the estimation of vector disparity. We have developed two different alternatives: a gradient-based one and a phase-based one. As mentioned, there are multiple examples for the horizontal disparity, while in the case of the vector disparity, as far as we know, the developments of this phase-based estimation in the literature are very seldom (see [13,17]), and their hardware implementation is even rarer. We have also designed a multi-scale generalization to increase the number of disparity levels 30× (using 5 spatial scales).

The requirements of a real-time system have been successfully fulfilled, since we are able to reach a working frequency of 32 fps with a VGA resolution (640 × 480) and, resorting on a multi-scale architecture, we are able to cope with large disparities. In the best case, the mono-scalar version of the system may achieve up to 267 fps for the same resolution, which shows the maximum level of parallelism provided by an FPGA (in our case, a Xilinx Virtex4 XC4vfx100 device). We have also analyzed the accuracy and density of our designs showing competitive results. By comparing different techniques with proper benchmark sequences, the Lucas-Kanade algorithm (including the homogenization stage) is revealed as the best choice, showing optimal efficacy *vs.* efficiency trade-off for vector disparity computation.

Hence, we have also compared the hardware and software versions of the algorithms; this comparison shows a low degradation for our hardware implementation which is affordable taking into account the use of fixed-point arithmetic instead of floating-point one. We have also compared the phase-based and the gradient-based algorithms and summarize the resource utilization. With the gradient-based algorithm, the resource cost is about 23% less than in the case of the phase-based one comparing the multi-scalar versions and 37% for the mono-scalar version. In terms of maximum working frequency, the gradient-based system is 2.3 times faster than the phase-based one.

The last section in this work deals with the implementation of the control model for a binocular camera system inspired by the Hering’s Law. The control manages the vergence, version, and tilt angles of the system to modify the fixation point in order to focus on a target object using the real-time vector disparity computation. The easy integration with such a system and the low power consumption of the system [36] support the employment of the FPGA as well.

In the paper, we have also presented a fixation application and a tracking trajectory example for a real-world scenario. The implementation is tackled using the real-time vector disparity estimation, computed by the gradient-based algorithm. It consists of moving the cameras towards the position of an object of interest and afterwards, in moving the fixation point using the computed vector disparity to take advantage of the optimal computation that can be performed at the zero disparity plane. Once the proper fixation is ensured, we achieved a second experiment for a smooth pursuit movement towards an object of interest. As shown, we achieve fixating at the point of interest in approximately 0.8 s (it involves the gazing towards the object and the vergence control).

Finally, as future works, we will address the integration of the system on a robotic mobile platform for the implementation of real algorithms for autonomous navigation and scene mapping.

## Figures and Tables

**Table 1.** Disparity performance comparison (works sorted by date of publication). For vector disparity implementations, two PDS values are given: the first considers only 1-D displacement performance and the second takes into account that 2-D matching of the vector methods have a search region that is the squared of the 1-D ones.

	**Resolution**	**Frame rate (fps)**	**PDS (×10^6^)**	**Architecture**	**Algorithm**
Our phase-based mono-scale core	640 × 480	118	218/1,304	Xilinx V4 (36 MHz)	2D Phase-based
Our Lucas–Kanade mono-scale core	640 × 480	267	492/1,968	Xilinx V4 (82 MHz)	2D Lucas–Kanade
Our phase-based multi-scale system	640 × 480	32	1,887/7,122	Xilinx V4 (42 MHz)	2D Phase-based
Our Lucas–Kanade multi-scale system	640 × 480	32	1,132/4,528	Xilinx V4 (41 MHz)	2D Lucas–Kanade
Tomasi (2010) [30]	512 × 512	28	939	Xilinx V4 (42 MHz)	1D Phase-based
Chang (2010) [51]	352 × 288	42	273	UMC 90nm Cell	1D Semi-Census
Hadjitheofanous (2010) [31]	320 × 240	75	184	Xilinx V2 Pro	1D SAD
Jin (2010) [32]	640 × 480	230	4,522	Xilinx V5 (93.1 MHz)	1D Census Transform
Calderon (2010) [33]	288 × 352	142	2,534	Xilinx V2 Pro (174.2 MHz)	1D BSAD
Chessa (2009) [17]	256 × 256	7	59/236	QuadCore Processor	2D Energy-based Pop. coding
Georgoulas (2009) [28]	800 × 600	550	21,120	Stratix IV (511 MHz)	1D SAD
Ernst (2009) [52]	640 × 480	4.2	165	GeForce 8800	1D SGM
Han (2009) [53]	320 × 240	144	707	ASIC (150MHz)	1D SAD
Gibson (2008) [54]	450 × 375	6	65	G80 NVIDIA	1D SGM
Diaz (2006) [34]	1,280 × 960	52	1,885	Xilinx V2 (65 MHz)	1D Phase-based
Gong (2005) [55]	384 × 288	16	30–60	ATI Radeon x800	1D GORDP

**Table 2.** Horizontal disparity performances: PoBP(%) percentage of pixels where MAE >1 and the density between parentheses.

	**Phase-based**		**Lucas-Kanade**	

	**SW**	**HW**	**SW**	**HW**
Tsukuba	16.65 (88.05%)	13.76 (63.42%)	21.77 (83.89%)	9.00 (40.89%)
Sawtooth	10.82 (78.11%)	10.58 (45.56%)	27.66 (77.10%)	5.90 (48.79%)
Venus	8.37 (70.99%)	7.84 (38.69%)	18.07 (56.83%)	7.57 (41.78%)
Teddy	25.73 (70.46%)	27.06 (37.55%)	40.91 (66.20%)	25.25 (40.85%)
Cones	27.18 (87.20%)	48.32 (61.10%)	58.06 (75.52%)	40.65 (52.45%)

**Table 3.** Vector disparity performances: PoGP(%), defined as the percentage of pixels where AAE < 5 deg. and the density between parentheses.

	**Phase-based**		**Lucas–Kanade**	

	**SW**	**HW**	**SW**	**HW**
plane 0H 0V	70.04 (93.66%)	93.77 (91.84%)	67.57 (74.17%)	68.67 (65.88%)
plane 15H 15V	69.99 (67.15%)	73.27 (91.73%)	67.82 (62.55%)	68.82 (49.76%)
desktop00	82.30 (94.84%)	80.48 (93.76%)	79.61 (70.29%)	80.68 (62.97%)
desktop09	84.24 (93.19%)	86.74 (85.26%)	82.92 (70.16%)	82.99 (62.57%)

**Table 4.** Hardware resource utilization for the presented complete architecture using a Virtex-4 FX100 FPGA (XC4vfx100).

	**4 input LUTs (out of 84,352)**	**Slice Flip-Flops (out of 84,352)**	**Slices (out of 42,716)**	**DSP (160)**	**Block RAM (378)**	**Freq (MHz)**
1D Phase-based system + Rectification	47,109 (56%)	27,221 (32%)	32,678 (76%)	159 (99%)	88 (23%)	44
1D Lucas–Kanade system + Rectification	55,152 (65%)	35,360 (41%)	38,560 (90%)	154 (96%)	100 (26%)	42
2D Phase-based Disp. system	55,445 (65%)	40,597 (48%)	37,383 (88%)	107 (66%)	126 (33%)	42
2D Lucas–Kanade Disp. system	33,039 (39%)	28,123 (33%)	27,749 (65%)	50 (31%)	148 (39%)	41
